# Development of a Spontaneous HPV16 E6/E7-Expressing Head and Neck Squamous Cell Carcinoma in HLA-A2 Transgenic Mice

**DOI:** 10.1128/mbio.03252-21

**Published:** 2022-01-04

**Authors:** Shiwen Peng, Deyin Xing, Louise Ferrall, Ya-Chea Tsai, Richard B. S. Roden, Chien-Fu Hung, T.-C. Wu

**Affiliations:** a Department of Pathology, The Johns Hopkins University grid.471401.7grid.21107.35grid.471401.7grid.21107.35grid.471401.7 , Baltimore, Maryland, USA; b Department of Oncology, The Johns Hopkins University grid.471401.7grid.21107.35grid.471401.7grid.21107.35grid.471401.7 , Baltimore, Maryland, USA; c Department of Obstetrics and Gynecology, The Johns Hopkins University grid.471401.7grid.21107.35grid.471401.7grid.21107.35grid.471401.7 , Baltimore, Maryland, USA; McMaster University

**Keywords:** human papillomavirus, HPV16, E6, E7, OPSCC, oral tumor model

## Abstract

Human papillomavirus (HPV)-associated head and neck squamous cell carcinoma (HNSCC) is a growing global health problem. HPV16 has been attributed to a majority of HPV-associated HNSCCs. In order to test candidate immunotherapies, we developed a spontaneous HPV16-driven HNSCC model in HLA-A2 (AAD) transgenic mice. We sought to eliminate the confounding effects of dominant HPV antigen presentation through murine major histocompatibility complex class I (MHC-I) via epitope mutagenesis (without compromising tumorigenicity). We generated HPV16 E6(R55K)(delK75) and E7(N53S) expression constructs with mutations in known dominant H-2D^b^ epitopes and characterized their presentation through murine and human MHC-I molecules using *in vitro* and *in vivo* activation of HPV16 E6/E7 antigen-specific CD8^+^ T cells. In addition, we tested the ability of E6(R55K)(delK75) and E7(N53S) for oncogenicity. The mutated E7(N53S) abolished the presentation of murine H-2D^b^-restricted HPV16 E7 peptide (i.e., amino acids [aa] 49 to 57) cytotoxic T lymphocyte (CTL) epitope and resulted in HLA-A2-restricted presentation of the HPV16 E7 (aa 11 to 20)-specific CTL epitope. The mutated E6(R55K)(delK75) abolished the activation of murine MHC-I-restricted E6-specific CD8^+^ T cell-mediated immune responses in C57BL/6 mice. In addition, the vaccination led to the activation of human HLA-A2-restricted E6-specific CD8^+^ T cell-mediated immune responses in HLA-A2 (AAD) transgenic mice. Injection of DNA plasmids encoding LucE7(N53S)E6(R55K)(delK75), AKT, c-Myc, and SB100 followed by electroporation results in development of squamous cell carcinoma in the oral/pharyngeal cavity of all of the HLA-A2 (AAD) transgenic mice (5/5), with 2/5 tumor-bearing mice developing metastatic carcinoma in the neck lymph nodes.

## INTRODUCTION

Head and neck squamous cell carcinoma (HNSCC) is a major global health problem, with more than 600,000 cases reported annually (1). Human papillomavirus (HPV) is detected in ∼65% of all HNSCC cases in the United States (2–6), and HPV-positive oropharyngeal squamous cell carcinoma (OPSCC) rates have been rising compared to HPV-negative OPSCC rates over the past 2 decades (2, 7). HPV16 is present in over 90% of HPV-associated OPSCCs. The identification of HPV16 as the primary etiological factor in a subset of HNSCCs has created the opportunity for development of HPV16-targeting immunotherapies to treat or prevent HPV-associated HNSCCs. Therapeutic HPV vaccines targeting HPV16 E6 and/or E7 viral proteins hold particular promise against established HPV-associated infection because HPV E6 and E7 are found in all HPV-associated malignancies, as they are functionally required for initiation and maintenance of cancer cells. Additionally, both E6 and E7 are non-“self” foreign antigens, which are not subject to central immune tolerance, and thus serve as promising targets for immunotherapies (8). However, in order to develop and evaluate the efficacy of an immunotherapeutic intervention in treatment of HPV-associated HNSCC, a suitable preclinical HPV^+^ tumor model is needed.

Such a preclinical HPV^+^ HNSCC tumor model should possess (i) the ability to form spontaneous HPV E6/E7-expressing HNSCCs, (ii) display carcinoma morphology, (iii) possess a local tumor microenvironment similar to that seen in patients, (iv) progress to an invasive and then metastatic state, (v) be applicable to different transgenic or knockout mice, and (vi) allow easy monitoring of tumorigenesis and disease burden/location. We previously developed a novel preclinical spontaneous tumor model of HPV16 E6/E7-expressing oral cancers in immunocompetent wild-type mice using oncogenic plasmids that express HPV16 E6/E7, luciferase, NRas^G12V^, and the Sleeping Beauty (SB) transposase (9, 10). Mice were injected with these plasmids into the submucosa of the oral/pharyngeal tract followed by local electroporation (EP). We used the SB system to drive integration of HPV16 oncogenes into the host genome, which results in stable, long-term E6/E7 and luciferase expression (9). We demonstrated that this novel procedure leads to integration of oncogenes at sites of plasmid EP, resulting in spontaneous tumorigenesis that can be monitored through luminescence imaging (9). However, the model still has major flaws, such as the generation of sarcoma instead of carcinoma morphology, which most human HPV-associated HNSCCs display.

In order to develop a preclinical model that is more suitable to human clinical translation, human major histocompatibility complex class I (MHC-I) transgenic mice were used. The employment of humanized MHC-I transgenic mice will potentially allow us to identify human MHC-I-restricted HPV-antigen specific cytotoxic T cell lymphocyte (CTL) epitopes. Such information would be very useful for the development of quantitative CD8^+^ T cell-mediated immunological assays and vaccine development. Furthermore, the development of a tumor model using human MHC-I transgenic mice will allow us to test therapeutic HPV vaccines capable of generating CD8^+^ T cells against HPV antigen presented by the human MHC-I molecules by tumor cells. Such information is critical for human translation. Currently, human leukocyte antigen-A2 (HLA-A2) is one of the most common MHC-I haplotypes (11). HLA-A*0201/D^d^ (AAD) transgenic mice express an interspecies hybrid class MHC-I gene, *AAD*, which contains the α-1 and α-2 domains of the human *HLA-A2.1* gene and the α-3 transmembrane and cytoplasmic domains of the mouse *H-2D^d^* gene/allele, under the direction of the human *HLA-A2.1* promoter (12). Thus, this transgenic strain allows us to model the human T cell immune responses to HLA-A2-presented antigens, which may be useful for testing immunotherapies for HPV-associated HNSCC. However, the strain also expresses the murine MHC-I molecules, such as H-2D^b^ and H-2K^b^ molecules. Thus, the potentially competitive effects of murine tumor antigen presentation must be considered when testing the HPV antigen-specific CD8^+^ T cell-mediated immune responses in HLA-A2 (AAD) transgenic mice.

We have recently shown that vaccination of HLA-A2 (AAD) transgenic mice with a DNA vaccine encoding calreticulin (CRT) linked to E7 (CRT/E7) (10) generated potent murine H-2D^b^-restricted HPV16 E7 peptide (amino acids [aa] 49 to 57)-specific CD8^+^ T cell-mediated immune responses instead of human HLA-A2-restricted E7-specific CD8^+^ T cell-mediated immune responses (13). Our data indicate that the presence of murine H-2D^b^-restricted E7-specific CTL epitope (aa 49 to 57) in the E7 gene of CRT/E7 DNA suppresses the presentation of HLA-A2-restricted E7-specific CTL epitopes (aa 11 to 20). Mutation of asparagine (N) 53 to serine (S) in E7 abolishes the presentation of the H-2D^b^-restricted E7 (aa 49 to 57) peptide-specific CTL epitope (14). In order to mitigate competition for the presentation of HLA-A2-restricted E7-specific cytotoxic CTL epitopes (e.g., aa 11 to 20) in HLA-A2 (AAD) transgenic mice, we tested a modified DNA vaccine construct encoding CRT linked to a mutated E7(N53S) gene: CRT/E7(N53S). We found that HLA-A2 (AAD) mice vaccinated with CRT/E7(N53S) DNA via *in vivo* EP elicited potent HLA-A2-restricted E7 peptide (aa 11 to 20)-specific CD8^+^ T cell-mediated immune responses (13), suggesting this mutation mitigated competition from H-2D^b^-restricted presentation.

Similarly, wild-type HPV16 E6 may also generate potent presentation of E6 through murine MHC-I molecules suppressing those mounted by human HLA-A2 presentation in HLA-A2 (AAD) transgenic mice. Thus, here we have constructed double mutant E6 by replacing the amino acid arginine (R) with lysine (K) at location 55 (R55K), with additional deletion of the lysine (K) at location 75 (delK75). We found that C57BL/6 mice vaccinated with DNA vaccine encoding CRT linked to mutated E6(R55K)(delK75) completely abolished the murine MHC-restricted HPV16 E6-specific CD8^+^ T cell-mediated immune responses. We further tested the mutated E7(N53S) and mutated E6(R55K)(delK75) for their ability to generate spontaneous oral/pharyngeal cancer using a similar approach, as we described previously (9, 13). We found that these mutated E6 and E7 genes, similar to wild-type E6 and E7 genes, were capable of generating spontaneous head and neck squamous cell carcinoma in HLA-A2 transgenic mice and potentially suitable for testing therapeutic HPV DNA vaccines. The potential utility of the newly created spontaneous HPV16 E6/E7-expressing HNSCC model in HLA-A2 (AAD) transgenic mice to test candidate therapeutic HPV16 vaccines is discussed.

## RESULTS

### Mutated HPV16 E7(N53S) reveals presentation of human MHC-I-restricted E7 peptide (aa 11 to 20)-specific CTL epitope through human HLA-A2 molecule in E7 DNA-transfected cells.

**FIG 1:**
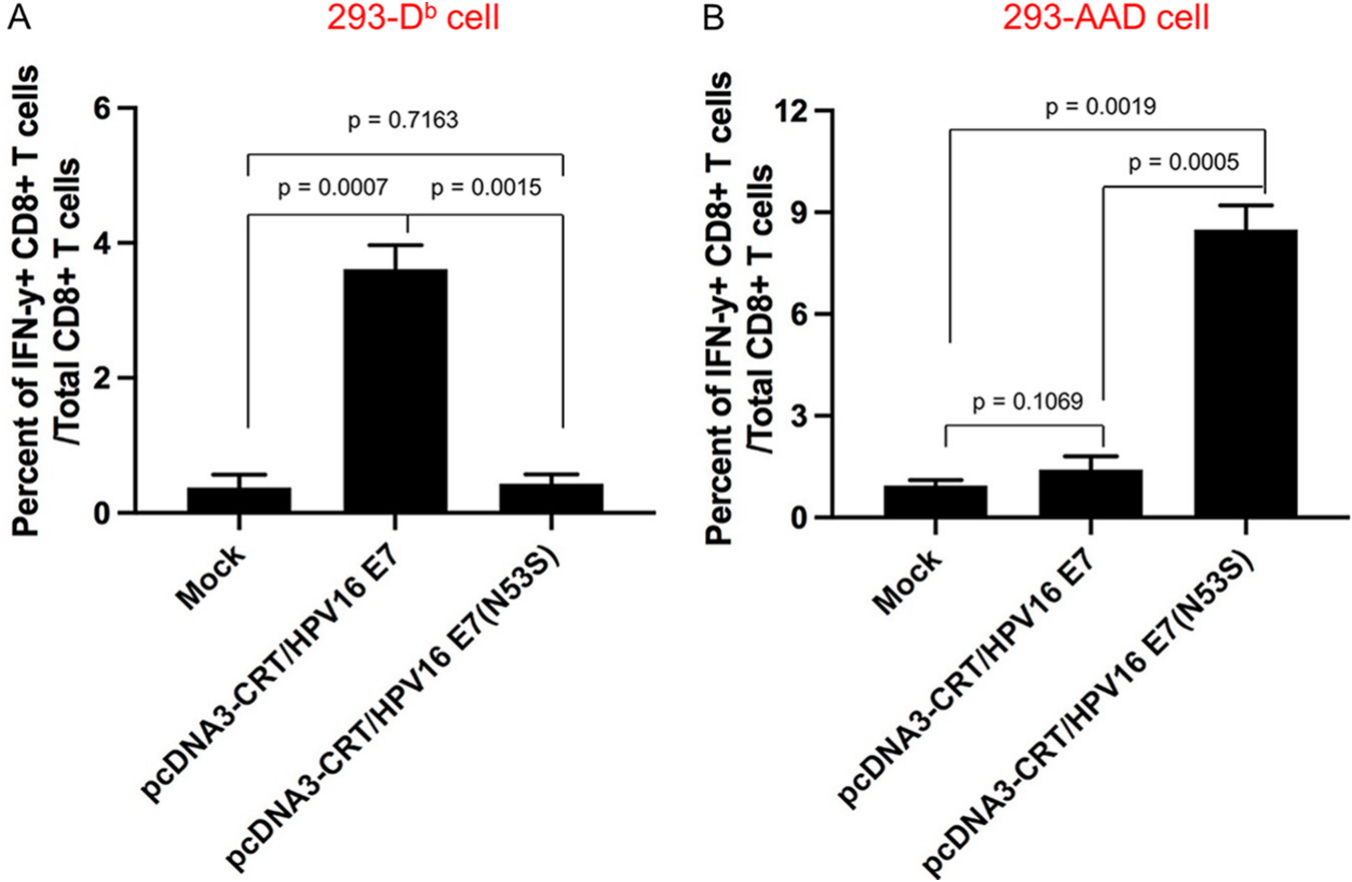
Comparison of CD8^+^ T cell epitope presentation between wild-type and mutant HPV16 E7(N53S) using 293 cells. 293-D^b^ or 293-AAD cells were transfected with either pcDNA3-CRT/HPV16 E7 or pcDNA-CRT/HPV16E7(N53S) and then were cocultured with either HPV16 E7 (aa 49 to 57) peptide- or HPV16 E7 (aa 11 to 20) peptide-specific CD8^+^ T cells in the presence of brefeldin A. Peptide-specific CD8^+^ T cell activation was analyzed by intracellular IFN-γ staining. (A) Summary of HPV16 E7-specific CD8^+^ T cell activation by 293-D^b^ cells by intracellular IFN-γ staining. (B) Summary of HPV16 E7-specific CD8^+^ T cell activation by 293-AAD cells by intracellular IFN-γ staining. Data are expressed as the mean percentage of IFN-γ^+^ CD8^+^ T cells out of the total number of CD8^+^ T cells ± SD.

**FIG 2:**
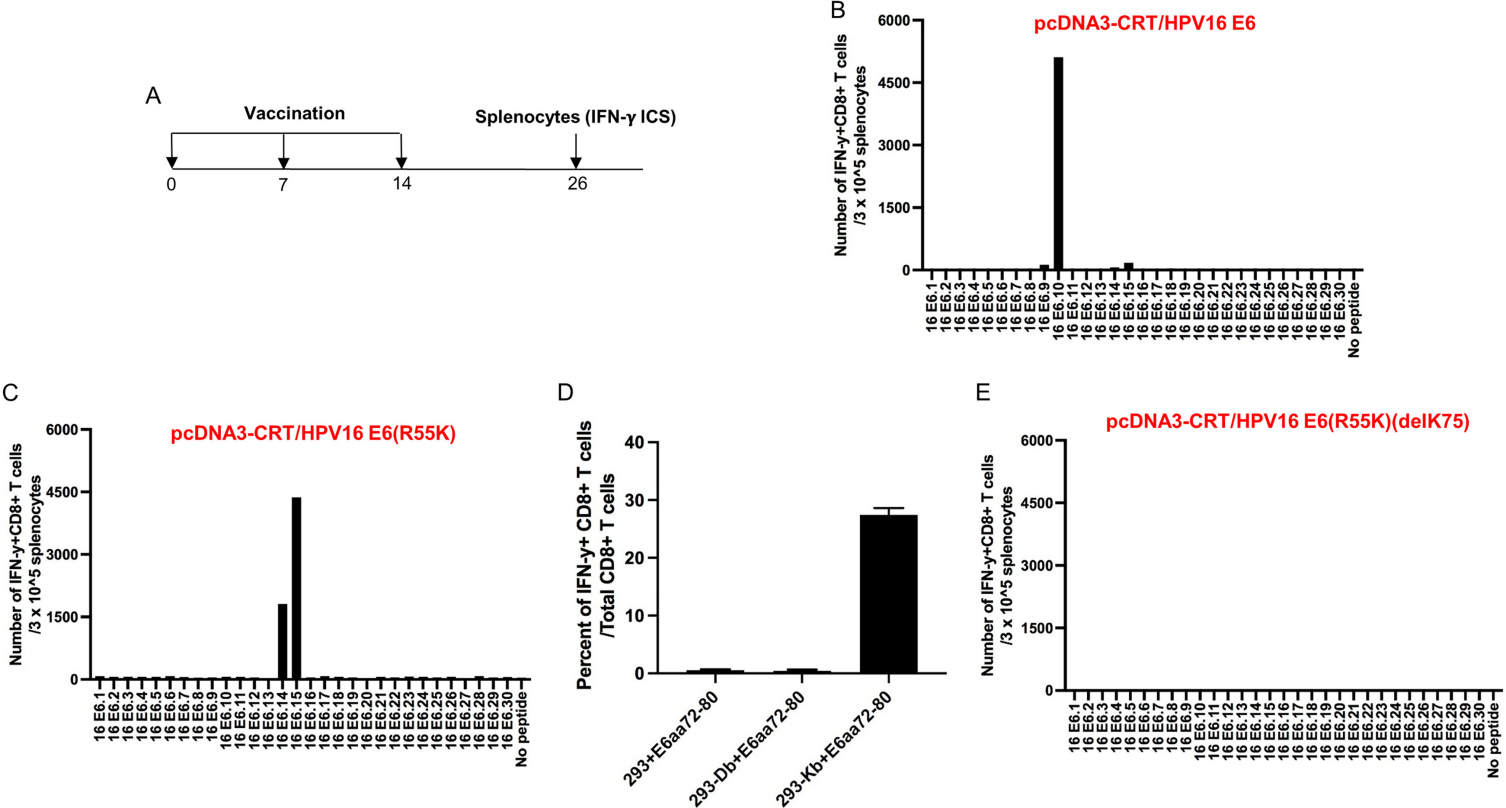
Characterization of HPV16 E6-specific CD8^+^ T cell-mediated immune responses in C57BL/6 mice vaccinated with different DNA constructs and determination of MHC-I restriction of HPV16 E6 (aa 72 to 80) peptide. (A) Schema of the experimental regimen. Briefly, female C57BL/6 mice (5 per group) were vaccinated with 20 μg/mouse of pcDNA3-CRT/E6, pcDNA3-CRT/E6(R55K), or pcDNA3-CRT/E6(R55K)(delK75) DNA vaccine on day 0 through intramuscular injection followed by electroporation. One week later, the mice were boosted once with the same dose and regimen. One week later, the vaccinated mice were boosted with TA-HPV vaccine by skin scarification. Twelve days after the TA-HPV vaccination, the splenocytes from the mice were prepared and stimulated with HPV16 E6 overlapping peptides that span the full-length of the HPV16 E6 protein or HPV16 E6 (aa 72 to 80) peptides. The cells were then stained with PE-conjugated anti-mouse CD8a. After permeabilization and fixation, the cells were stained with FITC-conjugated anti-mouse IFN-γ. The stained cells were analyzed by flow cytometry. (B to D) Bar graphs summarizing the numbers of activated (IFN-γ^+^) HPV16 E6-specific CD8^+^ T cell responses by the various peptides with splenocytes from C57BL/6 mice after vaccination with (B) pcDNA3-CRT/E6 DNA, (C) pcDNA3-CRT/E6(R55K) DNA, or (E) pcDNA3-CRT/E6(R55K)(delK75) DNA. (D) Determination of MHC-I restriction of HPV16 E6 (aa 72 to 80) CD8^+^ T cell epitope.

HLA-A2 (AAD) transgenic mice express both human MHC-I HLA-A2 and murine MHC-I molecules. We found that HLA-A2 (AAD) mice vaccinated with a DNA vaccine encoding CRT-linked wild-type E7 (CRT/E7) predominantly developed the murine H-2D^b^-restricted HPV16 E7 (aa 49 to 57)-specific CD8^+^ T cell response (13). Because the change of one amino acid from N to S abolishes H-2D^b^-restricted presentation of E7 CTL epitope aa 49 to 57, we vaccinated HLA-A2 (AAD) mice using CRT/E7(N53S) DNA vaccine followed by EP. These mice mounted a strong human HLA-A2-restricted HPV16 E7 peptide (aa 11 to 20)-specific CD8^+^ T cell-mediated immune response (13). Here, we further confirmed that the mutated E7(N53S) prevents murine MHC-I (H-2D^b^) presentation. Briefly, 293 cell lines were transfected with either murine MHC-I (H-2D^b^) or human MHC-I [HLA-A2 (AAD)] molecules to generate the H-2D^b^-expressing 293 cell line (293-D^b^) or HLA-A2 (AAD)-expressing 293 cell line (293-AAD). These cell lines were further transfected with CRT/E7 or CRT/E7(N53S) DNA constructs. Then they were incubated with either an H-2D^b^-restricted HPV16 E7 peptide (aa 49 to 57)-specific CD8^+^ T cell line or an HLA-A2-restricted HPV16 E7 peptide (aa 11 to 20)-specific CD8^+^ T cell line. As shown in Fig. 1A, H-2D^b^-expressing 293 cells transfected with CRT/E7 but not CRT/E7(N53S) DNA were able to significantly activate the H-2D^b^-restricted HPV16 E7 peptide (aa 49 to 57)-specific CD8^+^ T cells. Transfection of human HLA-A2 (AAD)-expressing 293 cells with CRT/E7(N53S) activated the HLA-A2-restricted HPV16 E7 peptide (aa 11 to 20)-specific CD8^+^ T cell line, but the CRT/E7 construct did not (Fig. 1B). These data indicated transfection of cells with DNA encoding CRT linked to E7(N53S) abolishes the presentation of E7 through the murine MHC-I (H-2D^b^) molecule, leading to the presentation of E7 through the human MHC-I (HLA-A2) molecule.

### HPV16 E6(R55K)(delK75) mutations eliminate murine MHC-I-restricted E6-specific CD8^+^ T cell-mediated immune response in C57BL/6 mice.

Using wild-type C57BL/6 mice, we next set out to characterize the murine MHC-I-restricted E6-specific CD8^+^ T cell-mediated immune responses. Female C57BL/6 mice were vaccinated with CRT/E6 DNA followed by EP (Fig. 2A). At 2 weeks following the final vaccination, splenocytes were harvested and prepared for analysis. Mice vaccinated with CRT/E6 DNA displayed a strong HPV16 E6.10 peptide-specific CD8^+^ T cell response (Fig. 2B). The E6.10 peptide contains a known murine H-2K^b^-restricted E6 peptide (aa 48 to 57)-specific CTL epitope (15). In order to eliminate the presentation of E6 protein through the H-2K^b^ molecule, we generate a mutated HPV16 E6, E6(R55K). Mice vaccinated with CRT/E6(R55K) DNA vaccine followed by EP failed to mount E6-specific CD8^+^ T cell-mediated immune responses against E6.10 peptide but created new strong CD8^+^ T cell-mediated immune responses against HPV16 E6.14 and E6.15 peptides (Fig. 2C). Further characterization of these immune responses indicated that these mice generated a strong HPV16 E6 peptide (aa 72 to 80)-specific CD8^+^ T cell-mediated immune response, which was present in both E6.14 and E6.15 overlapping peptides (Fig. 2D). We then generated the double mutant E6(R55K)(delK75). Mice vaccinated with CRT/E6(R55K)(delK75) DNA vaccine followed by EP were unable to mount any HPV16 E6-specific CD8^+^ T cell-mediated immune responses in vaccinated C57BL/6 mice (Fig. 2E). These data suggest that vaccination with DNA encoding CRT linked to E6(R55K)(delK75) fails to elicit murine MHC-I-restricted E6-specific CD8^+^ T cell-mediated immune responses in the C57BL/6 mice.

### Vaccination of HLA-A2 (AAD) transgenic mice with DNA encoding CRT linked to E6(R55K)(delK75) followed by boost with TA-HPV vaccinia generates potent HLA-A2-restricted E6 peptide (aa 29 to 38)-specific CD8^+^ T cell-mediated immune responses.

**FIG 3:**
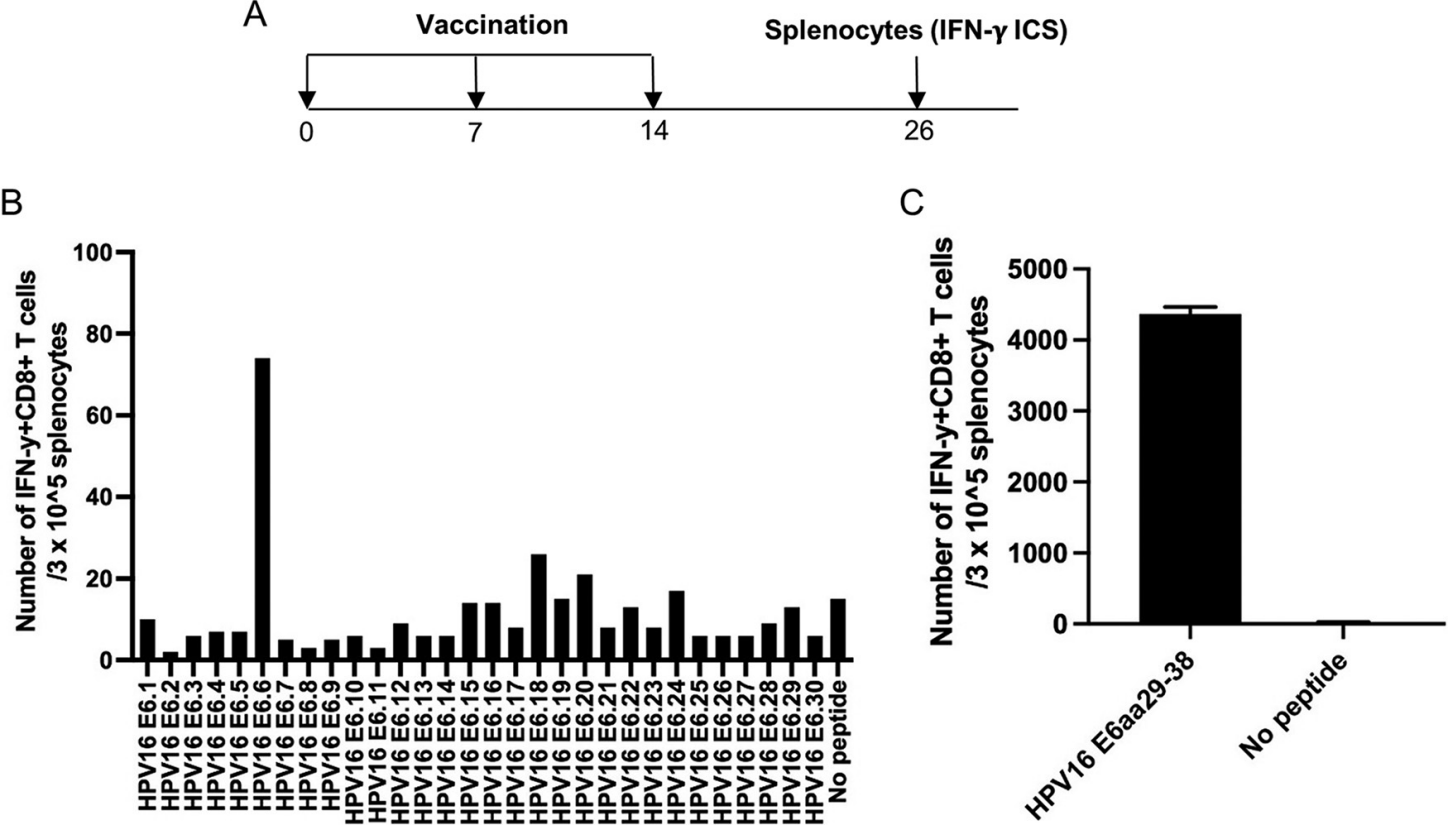
Characterization of HPV16 E6-specific CD8^+^ T cell mediated immune responses in HLA-A2 (AAD) transgenic C57BL/6 mice vaccinated with the pcDNA3-CRT/E6(R55K)(delK75) DNA construct. (A) Schema of the experimental regimen. Briefly, female AAD mice (5 per group) were vaccinated with 20 μg/mouse of pcDNA3-CRT/E6(R55K)(delK75) DNA vaccine on day 0 through intramuscular injection followed by electroporation. One week later, the mice were boosted once with the same dose and regimen. One week later, the vaccinated mice were boosted with TA-HPV vaccine by skin scarification. Twelve days after the TA-HPV vaccination, the splenocytes from the mice were prepared and stimulated with HPV16 E6 overlapping peptides that span the full length of the HPV16 E6 protein or HPV16 E6 peptides (aa 29 to 38). The cells were then stained with PE-conjugated anti-mouse CD8a. After permeabilization and fixation, the cells were stained with FITC-conjugated anti-mouse IFN-γ. The stained cells were analyzed by flow cytometry. (B) Bar graph summarizing the number of activated (IFN-γ^+^) HPV16 E6-specific CD8^+^ T cell responses in vaccinated AAD mouse splenocytes against HPV16 E6 overlapping peptides. (C) Bar graph summarizing the number of activated (IFN-γ^+^) HPV16 E6-specific CD8^+^ T cell responses in vaccinated AAD mouse splenocytes against HPV16 E6 (aa 29 to 38) peptide.

We reasoned that if the double mutant E6 is used in humanized HLA-A2 (AAD) transgenic mice, which still express murine MHC-I, the presentation of HPV16 E6 through murine MHC-I would not be a concern, and all E6 presentation detected and immune responses mounted should be resultant from human HLA-A2 presentation. In order to test this, we vaccinated HLA-A2 (AAD) mice with pcDNA3-CRT/E6(R55K)(delK75) DNA followed by EP twice at 1-week intervals. The DNA-vaccinated mice were boosted with TA-HPV vaccinia 1 week later (Fig. 3A). Vaccinations with TA-HPV alone have previously failed to generate detectable HPV antigen-specific CD8^+^ T cell-mediated immune responses (16). As shown in Fig. 3B, splenocytes derived from vaccinated mice generated potent HPV16 E6-specific CD8^+^ T cell-mediated immune responses against HPV16 E6.6 peptide. It was reported previously that HPV16 E6 protein contains an HLA-A2-restricted E6-specific CTL epitope (aa 29 to 38) (17, 18), and it is present in the HPV16 E6.6 peptide. To further confirm that the vaccinated HLA-A2 (AAD) mice generated potent HLA-A2-restricted E6 epitope (aa 29 to 38)-specific CD8^+^ T cell-mediated immune responses, we performed intracellular cytokine staining followed by flow cytometry analysis. As shown in Fig. 3C, splenocytes derived from vaccinated mice generated potent HPV16 E6-specific CD8^+^ T cell-mediated immune responses against HPV16 E6 (aa 29 to 38) peptide. Taken together, our data indicated that HLA-A2 (AAD) transgenic mice vaccinated with DNA vaccine encoding CRT linked to E6(R55K)(delK75) and boosted with TA-HPV generated only HLA-A2-restricted (but not murine MHC-I-restricted) E6-specific CD8^+^ T cell-mediated immune responses, even though TA-HPV expresses wild-type HPV16 E6.

### Injection of DNA plasmids encoding LucE6(R55K)(delK75)/E7(R53S), NRas^G12V^, and SB100 followed by EP results in development of sarcoma in the oral/pharyngeal cavity in HLA-A2 (AAD) transgenic mice.

**FIG 4:**
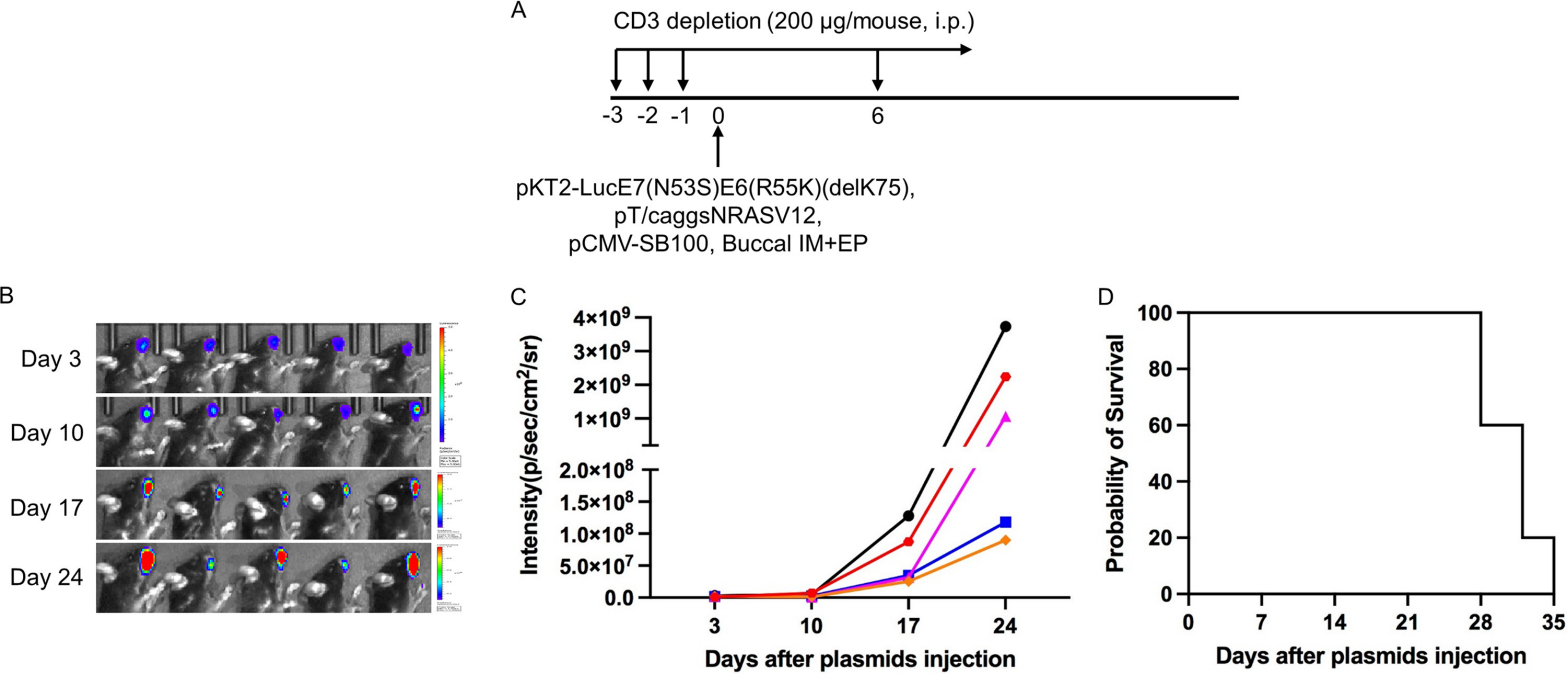
Generation of spontaneous HPV16 E6E7-expressing oral sarcoma model in HLA-A2 (AAD) transgenic mice. (A) Schema of the experiment. Briefly, 6- to 8-week-old female HLA-A2 [(HLA-A*0201/D^b^) (AAD)] mice (*n* = 5) were depleted of CD3^+^ T cells by daily intraperitoneal (i.p.) injection of purified anti-mouse CD3 monoclonal antibody (clone 17A2; 150 μg/mouse) for 3 days. One day later, the mice were injected with plasmids encoding LucE6(R55K)(delK75)/E7(R53S), NRas^G12V^, and Sleeping Beauty (SB) transposase through submucosa of the oral/pharyngeal cavity followed by electroporation (EP). The expression of luciferase (tumor growth signal) was monitored by bioluminescence imaging. (B) Bioluminescence image of tumor growth caused by plasmids injected into mice. (C). Summary line graph of the bioluminescence image. Each line represents growth in one mouse. (D). Summary Kaplan-Meier survival curve for Luc-HPV16E6(R55K)(delK75)/E7(N53S)-expressing tumor-bearing AAD mice.

**FIG 5:**
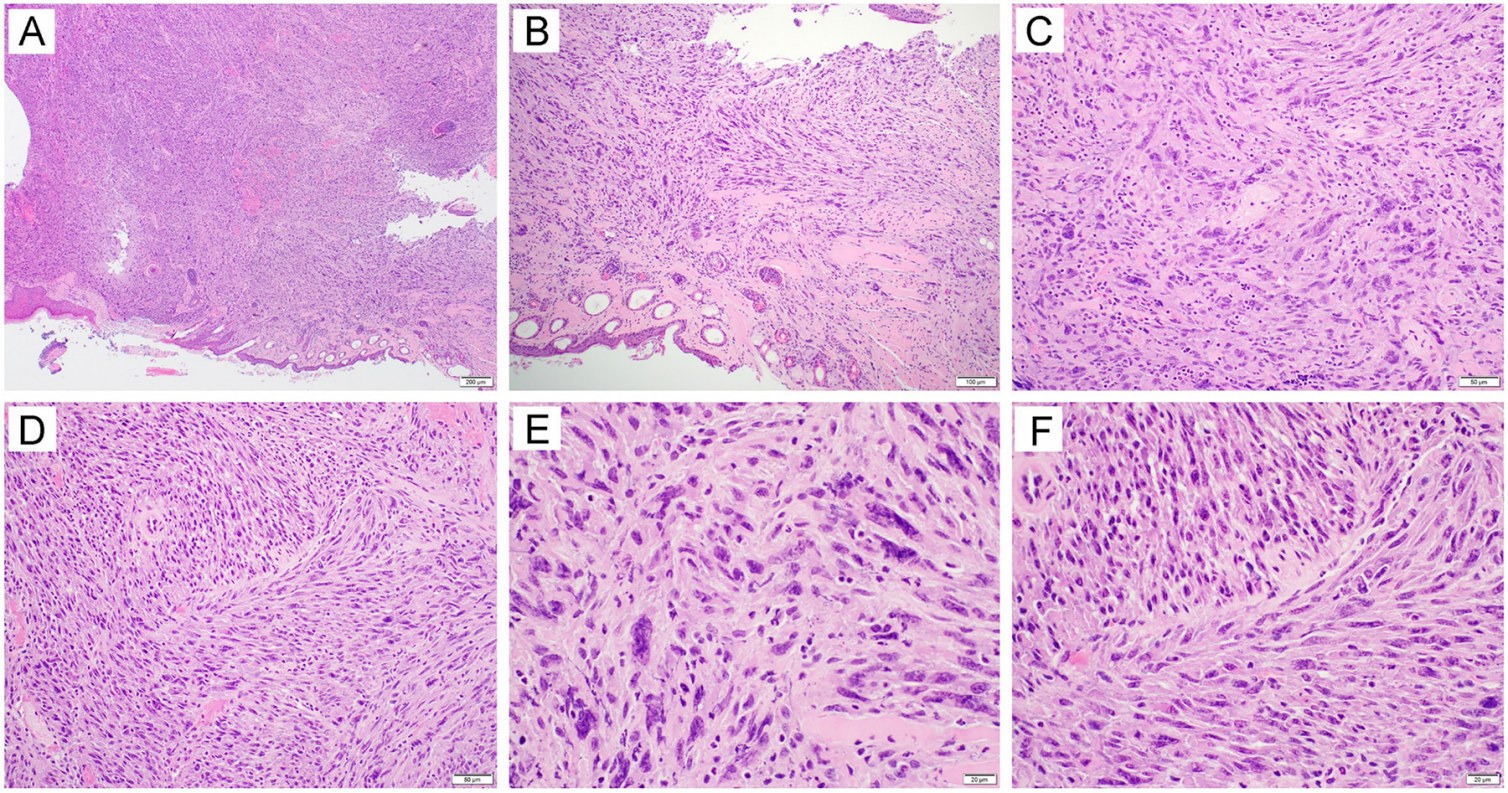
Histological examination of tumors formed in HLA-A2 (AAD) transgenic mice injected with plasmids encoding LucE6(R55K)(delK75)/E7(R53S), NRas^G12V^, and Sleeping Beauty transposase (SB100) followed by electroporation. Briefly, 6- to 8-week-old female HLA-A2 [(HLA-A*0201/D^d^) (AAD)] mice (*n* = 5) were depleted of CD3^+^ T cells by daily intraperitoneal injection of purified anti-mouse CD3 monoclonal antibody (clone 17A2; 150 μg/mouse) for 3 days. One day later, the mice were injected with plasmids encoding LucE6(R55K)(delK75)/E7(R53S), NRas^G12V^, and SB100 through submucosa of the oral/pharyngeal cavity followed by electroporation. At week 6, mice were sacrificed, and oral tissues were harvested for histological examination. The sections showed a mass lesion (A [40×]) with entrapment of hair follicles and skeletal muscle (B [100×]). The tumor was characterized as a malignant spindle cell proliferation arranged in disordered fascicles (C [200×]) with a focal storiform growth pattern (D [200×]). The tumor cells displayed significant cytologic atypia and brisk mitotic activity (E and F [400×]).

While mutant E6(R55K)(delK75) and mutant E7(N53S) were not presented through murine MHC-I molecules, one concern for using these E6/E7 mutants for the creation of the spontaneous E6/E7-expressing cancer in the oral/pharyngeal cavity is the loss of their oncogenicity. To generate spontaneous HPV16 E6/E7-expressing cancer in the oral/pharyngeal cavity in HLA-A2 (AAD) mice, mice were transiently depleted of CD3 by daily intraperitoneal injection of anti-CD3 monoclonal antibody once a day for 3 days. Then, DNA plasmids encoding LucE6(R55K)(delK75)/E7(R53S), NRas^G12V^, and SB100 were injected into the submucosa of the oral/pharyngeal cavity of mice followed by EP. The mice were observed for tumor development via bioluminescence imaging to detect the luciferase reporter (Fig. 4A). Of note, although we did not measure direct expression of HPV16 E6/E7 in the tumor of the oral/pharyngeal cavity, luciferase is a reliable surrogate marker for the expression of E6 and E7 because they are coexpressed by the same plasmid (9). By day 17 post-oncogenic plasmid injection and EP, most mice displayed some tumor growth, and the tumors continued to grow through day 24 (Fig. 4B and C). All mice (100%) were euthanized due to tumor burden by day 35 post-plasmid injection and EP (Fig. 4D). However, whereas most HPV-associated human oral/pharyngeal cancers display carcinoma morphology, the tumors generated via this procedure mainly displayed a sarcoma morphology. Under low-power magnification, the sections showed a mass (Fig. 5A) with entrapment of hair follicles and skeletal muscle (Fig. 5B). The tumor was characterized as a malignant spindle cell proliferation arranged in disordered fascicles (Fig. 5C) with a focal storiform growth pattern (Fig. 5D). The tumor cells displayed significant cytologic atypia and brisk mitotic activity (Fig. 5E and F). Our data indicated that despite the double mutations on E6 and single mutation of E7, they remain oncogenic and demonstrate the ability to generate cancer in the oral/pharyngeal cavity when combined with NRas^G12V^ and SB100.

### Injection of DNA plasmids encoding LucE7(N53S)E6(R55K)(delK75), AKT, c-Myc, and SB100 followed by EP results in development of squamous cell carcinoma in the oral/pharyngeal cavity in HLA-A2 (AAD) transgenic mice.

**FIG 6:**
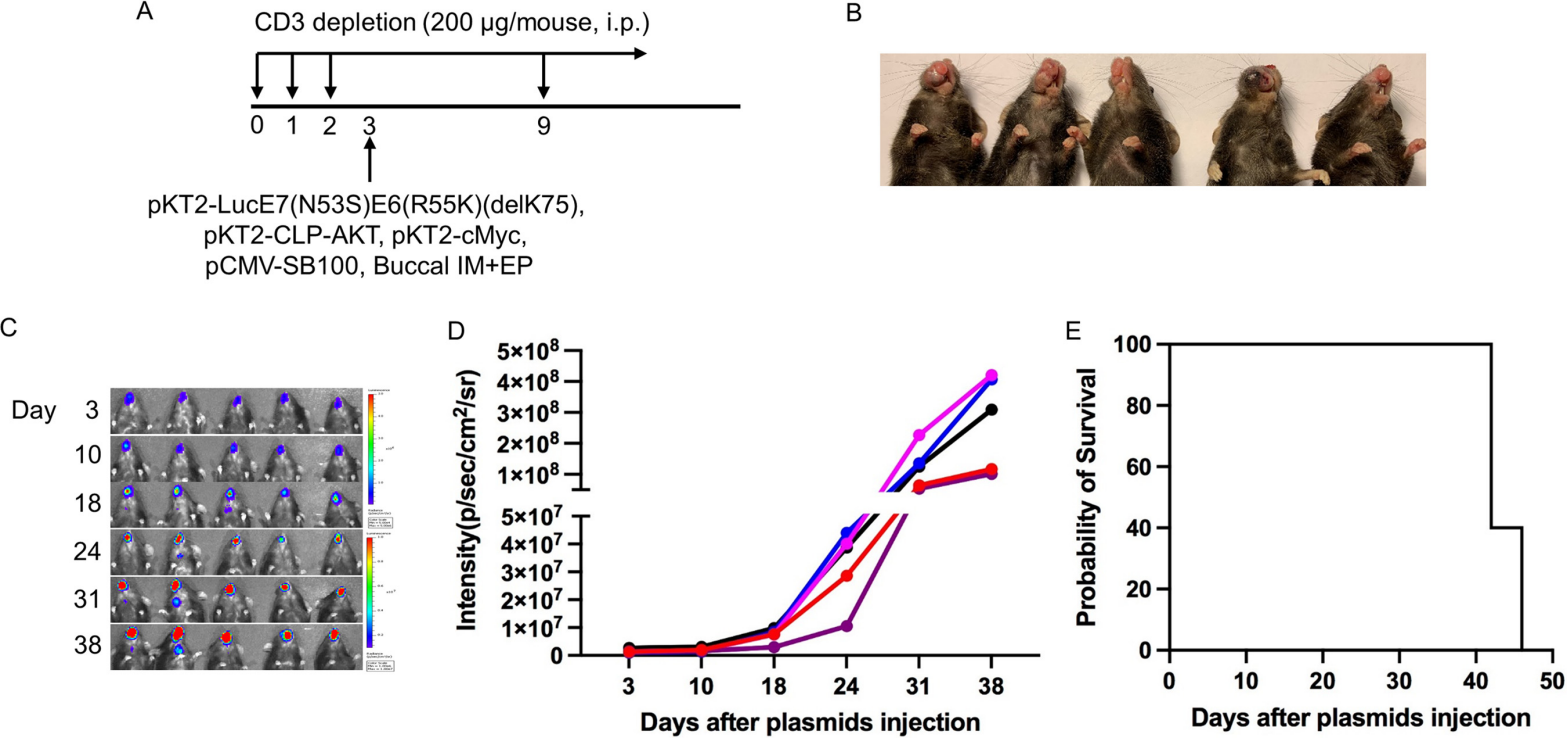
Generation of spontaneous HPV16 E6E7-expressing oral/pharyngeal carcinoma model in HLA-A2 (AAD) transgenic mice. (A) Schematic of the experiment. Briefly, 6- to 8-week-old female HLA-A2 [(HLA-A*0201/D^d^) (AAD)] mice (*n* = 5) were depleted of CD3^+^ T cells by daily intraperitoneal injection (i.p.) of purified anti-mouse CD3 monoclonal antibody (clone 17A2; 200 μg/mouse) for 3 days. One day later, the mice were injected with plasmids pKT2-LucE7(N53S)E6(R55K)(delK75), pKT2-CLP-AKT, pKT2-cMyc, and pCMV-SB100 via submucosal injection in the oral/pharyngeal cavity followed by electroporation (EP). (B) Gross image of oral/pharyngeal tumor induced by plasmids injection in AAD mice. (C) Bioluminescence image of tumor growth caused by plasmids injected into mice. (D) Summary line graph of the bioluminescence image. Each line represents growth in one mouse. (E) Survival curve of the oral tumor-bearing AAD mice.

**FIG 7:**
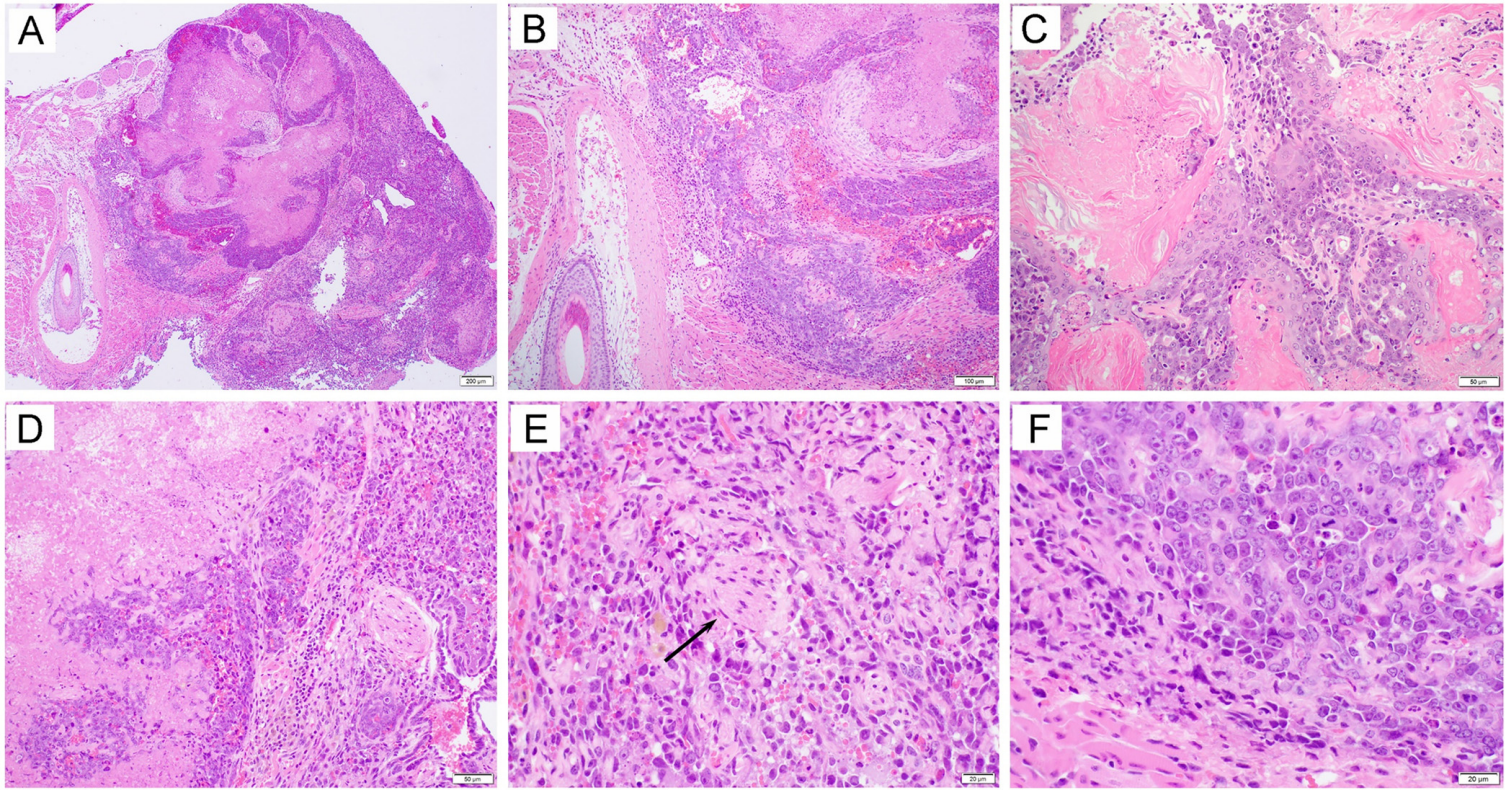
Histological examination of tumors formed in HLA-A2 (AAD) transgenic mice injected with plasmids encoding LucE7(N53S)E6(R55K)(delK75), pKT2-CLP-AKT, pKT2-cMyc, and pCMV-SB100 followed by electroporation. Briefly, 6- to 8-week-old female HLA-A2 [(HLA-A*0201/D^d^) (AAD)] mice (*n* = 5) were depleted of CD3^+^ T cells by daily intraperitoneal injection of purified anti-mouse CD3 monoclonal antibody (clone 17A2; 200 μg/mouse) for 3 days. One day later, the mice were injected with plasmids pKT2-LucE7(N53S)E6(R55K)(delK75), pKT2-CLP-AKT, pKT2-cMyc, and pCMV-SB100 via the submucosal injection in the oral/pharyngeal cavity followed by electroporation. At week 6 after oncogenic DNA transfection, mice were sacrificed and oral tissues were harvested for histological examination. The sections showed a nodular mass lesion (A [40×]) infiltrating into skeletal muscle (B [100×]). Extensive keratinization (C [200×]) and coagulative tumor cell necrosis (D [200×]) were present. On high-power magnification, the squamous cell carcinoma displayed epithelioid morphology, entrapped nerve tissue (E, arrow [400×]), and evident mitosis (F [400×]).

**FIG 8:**
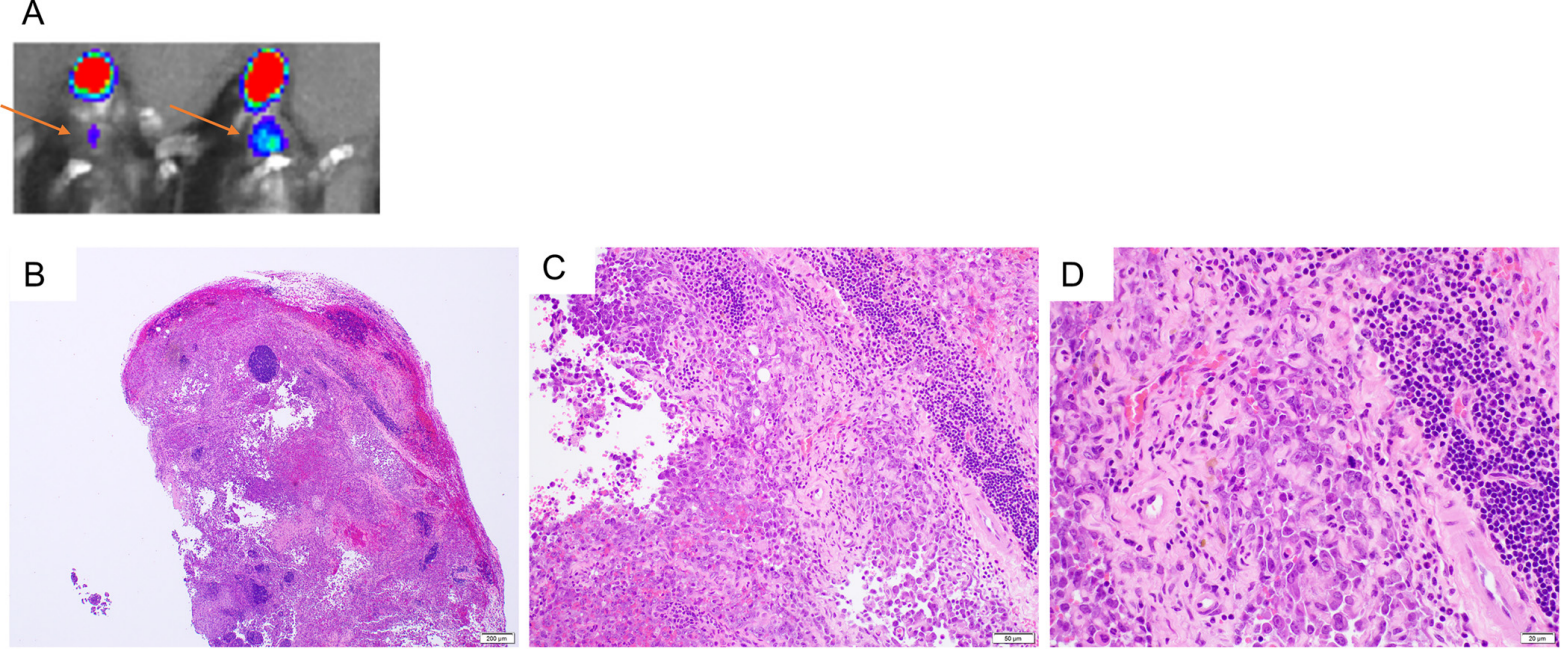
Histological examination of metastatic tumors of the neck lymph nodes derived from oral/pharyngeal carcinoma in HLA-A2 (AAD) transgenic mice injected with plasmids encoding LucE7(N53S)E6(R55K)(delK75), pKT2-CLP-AKT, pKT2-cMyc, and pCMV-SB100 followed by electroporation. Briefly, 6- to 8-week-old female HLA-A2 [(HLA-A*0201/D^d^) (AAD)] mice (*n* = 5) were depleted of CD3^+^ T cells by daily intraperitoneal injection of purified anti-mouse CD3 monoclonal antibody (clone 17A2; 200 μg/mouse) for 3 days. One day later, the mice were injected with plasmids pKT2-LucE7(N53S)E6(R55K)(delK75), pKT2-CLP-AKT, pKT2-cMyc, and pCMV-SB100 via submucosal injection in the oral/pharyngeal cavity followed by electroporation. At week 6 after oncogenic DNA transfection, mice were sacrificed, and the metastatic tumors in the neck lymph nodes were harvested for histological examination. (A) Representative luminescence imaging of tumor-bearing mice with neck metastasis. Arrows indicate the location of the neck metastasis. (B to D) Representative histological examination of the metastatic lymph node: B, 40×; C, 200×; D, 400×. Please note that the representative neck lymph node was largely replaced by metastatic squamous cell carcinoma.

We previously reported that the coexpression, in combination with HPV16 E6/E7, of AKT and c-Myc plasmids in the generation of a cervicovaginal tumor model results in carcinoma morphology (19). In order to determine whether the use of AKT and c-Myc would similarly result in carcinoma in our oral/pharyngeal HPV16 E6/E7-expressing tumor model, mice were transiently depleted of CD3. Then, DNA plasmids encoding LucE7(N53S)E6(R55K)(delK75), AKT, c-Myc, and SB100 were injected into the submucosa of the oral/pharyngeal cavity of mice, followed by EP (Fig. 6A). The mice were similarly followed for tumor development via bioluminescence imaging (Fig. 6C). All mice had begun to develop oral/pharyngeal tumor growth by day 18 (Fig. 6B and D). The tumors additionally resulted in the death of all mice by day 45 post-plasmid injection and EP (Fig. 6E). Of note, the oral/pharyngeal tumors had squamous cell carcinoma morphology. The sections showed a nodular mass lesion (Fig. 7A) infiltrating into skeletal muscle (Fig. 7B). Extensive keratinization (Fig. 7C) and coagulative tumor cell necrosis (Fig. 7D) were present. On high power, the squamous cell carcinoma displayed epithelioid morphology, entrapped nerve tissue (Fig. 7E, arrow), and evident mitosis (Fig. 7F). Furthermore, two of the five tumor-bearing mice developed a neck mass. As shown in Fig. 8, metastatic carcinomas were identified in the neck lymph nodes of the mice bearing carcinoma in the oral/pharyngeal cavity. Taken together, our data indicate that the use of AKT and c-Myc in our HPV16 oral tumor model results in tumor formation with carcinoma morphology.

## DISCUSSION

Here, we described that the N53S mutation of HPV16 E7 and the (R55K)(delK75) mutations of HPV16 E6 eliminated the presentation of E6 and E7 through murine MHC-I molecules. In addition, we demonstrated that submucosal injection of the DNA encoding mutated E6 and mutated E7 in combination with DNA encoding NRas^G12V^ and SB100 at the oral/pharyngeal cavity followed by EP induced spontaneous oral/pharyngeal sarcomas in HLA-A2 (AAD) mice. Furthermore, we found that submucosal injection of the DNA encoding mutated E6 and mutated E7 in combination with DNA encoding AKT, c-Myc, and SB100 in the submucosa of the oral/pharyngeal cavity followed by EP induced a spontaneous squamous cell carcinoma in HLA-A2 (AAD) mice. Thus, we have successfully created an improved HPV16 E6/E7-expressing HNSCC model in HLA-A2 (AAD) mice that eliminates the concerns for presenting E6/E7 antigens through murine MHC-I molecules. Of note, there is a morphological discrepancy between our carcinoma model and HPV-associated OPSCCs in humans. Our model was capable of keratinizing (Fig. 7C), whereas HPV-associated OPSCCs display mainly a basaloid, nonkeratinizing morphology based on HPV infection of tonsillar crypts (20). However, the difference in histology does not limit the ability to detect HLA-A2-restricted HPV16 E6/E7 epitopes in the model.

In the current studies, we observed different oncogenes generated different pathological phenotypes (sarcoma versus carcinoma). Although the exact mechanisms of these oncogenic constructs in generating these pathological features are not fully understood, Ras oncogenes have been implicated to be associated with the epithelial to mesenchymal transition (21, 22). Its role in transforming infected cells to mesenchymal types may be an explanation for why the NRas^G12V^ plasmid contributes to the sarcoma histology. Regarding the carcinoma phenotype, we previously found that coadministration of c-*myc* and AKT plasmids results in cervicovaginal carcinoma when administered with HPV16 E6/E7-luciferase and SB100 in the cervicovaginal track (19). Thus, coadministration of HPV16 E6/E7-expressing plasmid with different oncogenic plasmids may result in different morphological phenotypes.

Similar strategies can potentially be applied to other human HLA transgenic mice, such as HLA-A1, -A11, -A24, -B7, or -B24. These other HLA transgenic mice still express murine MHC-I molecules, such as H-2D^b^ and H-2K^b^. Thus, the newly developed approach of using mutated HPV16 E6 and E7 can potentially be extended to other human MHC-I transgenic mice for the development of high-risk HPV E6/E7-expressing tumors and for the characterization of human MHC-I-restricted E6/E7-specific CTL epitopes. Overall, our approach can (i) form spontaneous, localized HPV oncogene-induced tumors, (ii) display carcinoma morphology, (iii) follow progression from normal tissue to invasive and/or metastatic states, (iv) be applicable to different transgenic mice, and (v) be easily monitored. Therefore, the newly created HPV-associated head and neck squamous cell carcinoma model potentially can be used to test for various biological interventions, including vaccines and immunotherapies.

In the current studies, we noticed that the HPV16 E6 and E7 genes containing point mutations remain oncogenic. One potential explanation is that these point mutations [HPV16 E6(R55K)(delK75) and HPV16 E7(N53S)] are not in the regions of E6 and E7 that are critical for the interaction of key cellular proteins, such as p53 or Rb, which are important for the carcinogenesis of HPV-associated malignancies (23–29). Furthermore, the N53S mutation on the E7 protein does not impact the HLA-A2-restricted E7-specific CTL epitopes (aa 11 to 20, aa 82 to 90, and aa 86 to 93) that were described previously (17, 30). Thus, the changes in these mutants do not alter the functions of the oncogenic proteins, and they are still capable of dysregulating the cell and causing tumor development. This further justifies the use of the mutated HPV16 E6 and E7 in different transgenic HLA mice as they only abolish murine MHC-I presentation of E6 and E7 and remain oncogenic.

Although mutating HPV16 E6/E7 enables us to analyze human HLA-A2-restricted CTL presentation, we recognize that the mutations may have altered natural human HLA-A2 CTL epitope presentation. Meaning, although the N53S mutation in the E7 mutant and the dual R55K and delK75 mutations in the E6 mutant allow us to eliminate the immunodominant murine epitopes, we may have erroneously deleted a CTL epitope that would have otherwise elicited an HLA-restricted E6/E7-specific CD8^+^ T cell response. For example, for E7 the N53S mutation may prevent our ability to identify a native peptide covering that mutation. To circumvent this limitation, future investigation should use synthetic 9-mers spanning the wild-type E7 at aa 53 (aa 45 to 53 through aa 53 to 61, with 8 amino acids overlapping for 9 peptides in total) for further characterization. Humanized transgenic MHC-I mice will receive a peptide vaccine with CpG adjuvant (31), and if the peptide elicits a CD8^+^ T cell response, we will confirm the endogenous processing of the peptide via incubation of antigen-specific CD8^+^ T cells with a live human MHC-I-expressing cell line transfected with the DNA encoding the specific 9-mer peptide using methods described previously (15, 32). A similar process with synthetic 9-mers spanning the wild-type E6 at aa 55 (aa 47 to 55 through aa 55 to 63, with 8 amino acids overlapping for 9 peptides in total) could be used for the E6(R55K) mutation. Furthermore, any wild-type peptide found to elicit a CD8^+^ T cell response will need to be confirmed for endogenous processing using methods described previously (15, 32).

The second potential limitation of the mutations is that they may create a new, artificial CTL epitope. Further investigations to determine the responses were not caused by an artificially produced CTL epitope due to the mutation or the deletion of the particular amino acid introduced by the DNA vaccines should be conducted. Synthetic overlapping 9-mer peptides covering the mutated E7(N53S) or E6(R55K) regions can be used to challenge the mice. If any of these peptides elicit a response when the corresponding peptide derived from wild-type E7/E6 does not, an artificial epitope may have been created. Due to the deletion mutation in E6 at aa 75 (delK75), to determine that the delK75 mutation did not create an artificial epitope, mice could be challenged also with synthetic 9-mers covering the mutated E6 (delK75) from aa 68 to aa 83. Again, if any of these peptides elicit a positive response when the corresponding wild-type E6 peptides does not, an artificial epitope may have been created by the deletion.

In summary, we have successfully developed a system that can be used for the characterization of human MHC-I-restricted E6/E7-specific CTL epitopes using different human MHC-I transgenic mice. Such information is critical for the development of quantitative immunological assays for the characterization of HPV E6/E7-specific CD8^+^ T cell-mediated immune responses. Such assays are necessary to assess the immunogenicity and potency generated by different candidate therapeutic HPV vaccines. Here, we describe a spontaneous HPV16 E6/E7-expressing head and neck squamous cell carcinoma in HLA-A2 (AAD) transgenic mice driven by the expression of mutated E6/E7. The model can also potentially be used for testing chemotherapeutic interventions for HPV-associated HNSCCs, including novel targeted small molecules, to assess their clinical translational potentials.

## MATERIALS AND METHODS

### Mice.

Five- to ∼8-week-old female C57BL/6 mice were purchased from Taconic Biosciences (Germantown, NY). HLA-A*0201/D^d^ (AAD) transgenic C57BL/6 mice (12) were kindly provided by Victor Engelhard at the University of Virginia Health Sciences Center and maintained at the Johns Hopkins University Sidney Kimmel Comprehensive Cancer Center animal facility. The transgenic mouse expresses a chimeric HLA class I molecule comprising the α-1 and α-2 domains of HLA-A*0201 and the α-3 transmembrane and cytoplasmic domain of H-2D^d^. All mice were maintained at the cancer center animal facility under specific-pathogen-free conditions at Johns Hopkins University School of Medicine (Baltimore, MD). All procedures were performed according to preapproved protocols and in accordance with recommendations for the proper use and care of laboratory animals.

### Plasmids.

pCMVΔR8.91, pMDG, and pCDH1-puro were described previously (33). The generation of pMSCV-HLA-A2 (AAD) was described in reference 34. The pCDH1-puro-AAD DNA construct was generated by first isolating the DNA fragment containing the HLA-A2 (AAD) gene from pMSCV-HLA-A2 (AAD) using XbaI and BamHI and further cloning into the XbaI and BamHI sites of pCDH1-puro. The generation of pcDNA3-CRT/16E7 (10) and pcDNA3-CRT/16E7(N53S) (13) were described previously. The generation of pcDNA3-CRT/16E6 was described previously (15).

The generation of pT/Caggs-NRasV12 plasmid and pKT2/CLP-AKT plasmid was described previously (35). The construction of the pCMV(CAT)T7-SB100 plasmid was described previously (36). These plasmids were purchased from Addgene. The generation of Pkt2-cMyc was described previously (19).

To generate pcDNA3-CRT/16E6(R55K), HPV16 E6(R55K) DNA was first amplified via PCR using the pcDNA3-CRT/16E6 template and the following set of primers: 5′-GGCCGAATTCATGCACCAAAAGAGAACTG-3′, 5′-GTTACTGCGACGTGAGGTATTTGAATTTGCTTTTAAGGATTTATGCATAGTATATA-3′, 5′-TATATACTATGCATAAATCCTTAAAAGCAAATTCAAATACCTCACGTCGCAGTAAC-3′, and 5′-GCCAAGCTTTTACAGCTGGGTTTCTCTAC-3′. The amplified PCR product was then cloned into the EcoRI/HindIII sites of pcDNA3-CRT. To generate pcDNA3-CRT/16E6(R55K)(delK75), 16E6(R55K)(delK75) DNA was first amplified via PCR using the pcDNA3-CRT/16E6 (R55K) template and the following set of primers: 5′-GGCCGAATTCATGCACCAAAAGAGAACTG-3′, 5′-GCTGTATGTGATAAATGTTTATTTTATTCTAAAATTAGTGAG-3′, 5′-CTCACTAATTTTAGAATAAAATAAACATTTATCACATACAGC-3′, and 5′-GCCAAGCTTTTACAGCTGGGTTTCTCTAC-3′. The amplified PCR product was then cloned into the EcoRI/HindIII sites of pcDNA3-CRT. To generate Pkt2-Luc-T2a-E7(N53S)-T2a-E6, E7(N53S) DNA was first amplified via PCR using the Pkt2-Luc-T2a-E7-T2a-E6 template and the following set of primers: 5′-AAACTCGAGGAGGGCAGAGGAAGTCTTCT-3′, 5′-AGAACCGGACAGAGCCCATTACAGTATTGTAACCTTTTGTTGCAAGTG-3′, 5′-CACTTGCAACAAAAGGTTACAATACTGTAATGGGCTCTGTCCGGTTCT-3′, and 5′-TTTGAATTCTGGTTTCTGAGAACAGATGG-3′. The amplified PCR product was then cloned into the XhoI/EcoRI sites of Pkt2-Luc-T2a-E7-T2a-E6. To generate Pkt2-Luc-T2a-E7(N53S)-T2a-E6 (R55K)(delK75), E6 (R55K)(delK75) DNA was first amplified via PCR using the pcDNA3-CRT/16E6(R55K)(delK75) template and the following set of primers: 5′-TTTGAATTCGAGGGCAGAGGAAGTCTTCT-3′, and 5′-TTTCCAGCTAGCTGGTTACAGCTGGGTTTCTCTACG -3′. The amplified PCR product was then cloned into the EcoRI/BstXI sites of Pkt2-Luc-T2a-E7(N53S)-T2a-E6. The accuracy of the newly generated DNA constructs was confirmed by DNA sequencing.

### Peptides, antibodies, and other reagents.

HPV16 E6 overlapping peptides (15 aa long, each with a 10-aa overlap), HPV16 E6 peptide (aa 29 to 38) TIHDIILECV and HPV16 E6 peptide (aa 72 to 80) KCLKFYSKI were synthesized by GenScript (Piscataway, NJ) at a purity of ≥80%. Phycoerythrin (PE)-conjugated anti-mouse CD8a (clone 53.6.7), fluorescein isothiocyanate (FITC)-conjugated anti-mouse gamma interferon (IFN-γ) (clone XMG1.2), FITC-conjugated anti-HLA-A*0201 (clone BB7.2) antibodies and brefeldin A were purchased from Biolegend (San Diego, CA). Purified anti-mouse CD3 monoclonal antibody (clone 17A2) was purchased from Bio X Cell (West Lebanon, NH). Lipofectamine 2000 was purchased from Invitrogen (Carlsbad, CA). Polybrene and puromycin were purchased from Sigma-Aldrich (St. Louis, MO). Recombinant mouse interleukin-2 (IL-2) protein was purchased from R&D Systems (Minneapolis, MN).

### Cell lines.

The establishment of HPV16 E6- and E7-expressing TC-1 cells was described previously (37). The generation of TC-1/A2 cells was also described previously (34). These cells were maintained in RPMI medium supplemented with 2 mM glutamine, 1 mM sodium pyruvate, 100 IU mL^−1^ penicillin, 100 μg mL^−1^ streptomycin, and 10% fetal bovine serum (FBS). The 293 cell line is a human embryonic kidney cell line and was purchased from ATCC (Manassas, VA). The 293 cell line expressing the simian virus 40 (SV40) T antigen, 293T, was also purchased from ATCC. The establishment of 293 cell lines expressing either murine MHC-I molecule K^b^ (38) or D^b^ (31) has been reported. To generate a 293 cell line expressing chimeric HLA-A*0201/D^d^ (293-AAD), 293T cells were transfected with pCDH1-puro-AAD, pCMVΔR8.91, and pMDG using Lipofectamine. Forty-eight hours after transfection, the virus-containing supernatant was collected, filtered, and used to transduce 293 cells in the presence of 8 mg/mL Polybrene. The HLA-A2 (AAD)-expressing 293 cells were selected with puromycin (5 μg/mL), and the expression of AAD was confirmed by flow cytometry analysis. 293-D^b^, 293-K^b^, and 293-AAD cells were cultured in Dulbecco’s modified Eagle medium (DMEM) containing 2 mM glutamine, 1 mM sodium pyruvate, 100 IU mL^−1^ penicillin, 100 μg mL^−1^ streptomycin, and 10% FBS. The establishment of the murine HPV16 E7 peptide (aa 49 to 57)-specific CD8^+^ T cell line (39) and the generation of the murine HPV16 E7 peptide (aa 11 to 20)-specific CD8^+^ T cell line (34) were described previously. These T cells were maintained by weekly restimulation with either irradiated TC-1 or TC-1/A2 cells in the presence of recombinant murine IL-2.

### *In vitro* HPV16 E7 antigen presentation assay.

To assess CD8^+^ T cell epitope presentation by either wild-type HPV16 E7 or mutant HPV16 E7(N53S), first we transfected 293-D^b^ cells with either pcDNA3-CRT/16E7 or pcDNA3-CRT/16E7(N53S) using Lipofectamine 2000. The transfected 293-D^b^ cells were harvested 24 h later and cocultured with HPV16 E7 peptide (aa 49 to 57)-specific CD8^+^ T cells (1:1 ratio) in a 96-well round-bottom plate in the presence of GolgiPlug (BD Pharmingen, San Diego, CA) for 20 to 24 h. Similarly, 293-AAD cells were transfected with either pcDNA3-CRT/16E7 or pcDNA3-CRT/16E7(N53S) using Lipofectamine 2000. The transfected cells were harvested 24 h later and cocultured with HPV16 E7 peptide (aa 11 to 20)-specific CD8^+^ T cells in the presence of brefeldin A (5 μg/mL). The presentation of H-2D^b^-restricted HPV16 E7 peptide (aa 49 to 57)-specific CD8^+^ T cell epitope or HLA-A*0201-restricted HPV16 E7 peptide (aa 11 to 20)-specific CD8^+^ T cell epitope was analyzed by the activation of either the HPV16 E7 peptide (aa 49 to 57)-specific or HPV16 E7 peptide (aa 11 to 20)-specific CD8^+^ T cell line using intracellular IFN-γ staining followed by flow cytometry analysis.

### Establishment of T cell line and determination of MHC-I restriction.

To establish the HPV16 E6 peptide (aa 72 to 80)-specific CD8^+^ T cell line, splenocytes from female C57BL/6 mice vaccinated with pcDNA3-CRT/HPV16 E6(R55K) DNA, followed by boost vaccination with TA-HPV by skin scarification at a dose of 5 × 10^4^ PFU/mouse (16) 1 week later, were harvested 1 week after the last vaccination. The splenocytes were stimulated with irradiated, HPV16 E6 peptide (aa 72 to 80)-loaded TC-1 cells in the presence of murine IL-2 (20 U/mL) and were restimulated once a week. To determine the MHC-I restriction element, 293, 293-D^b^, or 293-K^b^ cells were pulsed with HPV16 E6 peptide (aa 72 to 80) (5 μg/mL), followed by extensive washing with complete RPMI 1640 medium containing 10% FBS. These cells were then cocultured with HPV16 E6 peptide (aa 72 to 80)-specific CD8^+^ T cells (effector/target ratio of 4:1) in the presence of brefeldin A (5 μg/mL) overnight. The activation of HPV16 E6 peptide (aa 72 to 80)-specific CD8^+^ T cells was analyzed by IFN-γ intracellular cytokine staining assay.

### Vaccines and vaccination.

The DNA vaccines pcDNA3-CRT/16E6, pcDNA3-CRT/16E6(R55K), and pcDNA3-CRT/16E6(R55K)(delK75) were prepared by using an endotoxin-free kit (Qiagen, Valencia, CA). TA-HPV is a recombinant vaccinia virus expressing HPV16/18 E6/E7, and it was described previously (40). DNA was given to C57BL/6 mice via intramuscular (i.m.) injection followed by EP using an Electro Square Porator (Holliston, MA) at the hind leg muscle. The mice were boosted once 7 days later with the same regimen. Seven days after the second DNA vaccination, the mice were further boosted with TA-HPV via skin scarification, as described previously (41).

### Intracellular cytokine staining and flow cytometry analysis.

To analyze the activation of HPV16 E7 peptide (aa 49 to 57)- or (aa 11 to 20)-specific CD8^+^ T cells, the cells were stained for surface CD8 and intracellular IFN-γ followed by flow cytometry analysis. To detect HPV16 E6-specific CD8^+^ T cell responses by intracellular cytokine staining, 12 days after the last vaccination, splenocytes were prepared from vaccinated mice and stimulated with HPV16 E6 overlapping peptides (5 μg/mL) or short HPV16 E6 peptide (1 μg/mL) in the presence of brefeldin A (5 μg/mL) at 37°C overnight. The cells were then harvested, washed with PBS containing 0.5% bovine serum albumin (BSA), and stained with PE-conjugated anti-mouse CD8a. Cells were fixed and permeabilized using the Cytofix/Cytoperm kit according to the manufacturer’s instruction (eBioscience, San Diego, CA). FITC-conjugated anti-mouse IFN-γ was used to stain intracellular IFN-γ. Flow cytometry analysis was performed using FACSCalibur flow cytometer with CELLQuest software.

### Establishment of spontaneous oral/pharyngeal HPV16 E6/E7-expressing tumor model.

To establish the HPV16 E6/E7-expressing oral/pharyngeal tumor model using Ras oncogene, 5- to ∼8-week-old female HLA-A2 (AAD) transgenic mice were injected with anti-mouse CD3 monoclonal antibody (200 μg/mouse) through intraperitoneal injection for three continuous days. One day after the last injection, plasmids Pkt2-Luc-T2a-E7(N53S)-T2a-E6(R55K)(delK75), pT/Caggs-NRasV12, and pCMV(CAT)T7-SB100 were injected into the submucosa of the oral/pharyngeal cavity (10 μg/plasmid, 30 μL/injection), followed by EP with an Electro Square Porator (Holliston, MA), as described previously (13). To establish HPV16 E6/E7-expressing oral/pharyngeal tumor model using AKT and c-Myc oncogenes, all the procedures were the same as described above, except the following plasmids were used: Pkt2-Luc-T2a-E7(N53S)-T2a-E6 (R55K)(delK75), pKT2/CLP-AKT, Pkt2-cMyc, and pCMV(CAT)T7-SB100 (10 μg/plasmid, 30 μL/injection). Anti-mouse CD3 monoclonal antibody treatment was maintained once weekly. Tumor growth was monitored using bioluminescence imaging (Xenogen IVIS spectrum bioluminescence imaging series 2000; Alameda, CA), gross inspection, and histological examination. When the tumor in the oral/pharyngeal cavity grew too large, the mouse was unable to eat and began to lose weight. When the tumor-bearing mice lost more than 20% of their body weight compared to an untreated control mouse, the mice were euthanized according to protocols approved by the Johns Hopkins Animal Care and Use Committee. The tumor growth resulted in death, either by nature or euthanization, usually by day 35 for sarcoma formation and by day 49 for carcinoma formation post-oncoplasmid injection and EP.

### Histology.

Spontaneously formed tumors in the oral/pharyngeal cavity were surgically removed and placed into 10% neutral buffered formalin solution. The tumor tissues were then paraffin embedded, and hematoxylin and eosin (H&E) staining was performed. The histology slides were reviewed with consensus by two board-certified pathologists from the Department of Pathology (D. Xing and T.-C. Wu) in the Johns Hopkins University School of Medicine (Baltimore, MD).

### Statistical analysis.

Data are presented as means and standard deviations. Individual data points were compared by Student's *t* test. Survival of the tumor-bearing mice was estimated using the Kaplan-Meier survival estimator. A *P* value of <0.05 was considered significant. Statistical analysis was performed using Prism 9 software (GraphPad).

### Data availability.

All data and materials are available from the corresponding author upon written request.
