# Erratum for Peng et al., “Development of a Spontaneous HPV16 E6/E7-Expressing Head and Neck Squamous Cell Carcinoma in HLA-A2 Transgenic Mice”

**DOI:** 10.1128/mbio.00296-22

**Published:** 2022-03-01

**Authors:** Shiwen Peng, Deyin Xing, Louise Ferrall, Ya-Chea Tsai, Richard B. S. Roden, Chien-Fu Hung, T.-C. Wu

**Affiliations:** a Department of Pathology, The Johns Hopkins University grid.471401.7grid.21107.35 , Baltimore, Maryland, USA; b Department of Oncology, The Johns Hopkins University grid.471401.7grid.21107.35 , Baltimore, Maryland, USA; c Department of Obstetrics and Gynecology, The Johns Hopkins University grid.471401.7grid.21107.35 , Baltimore, Maryland, USA

## ERRATUM

Volume 13, issue 1, e03252-21, 2021, https://doi.org/10.1128/mbio.03252-21. Labels in Fig. 4A and 6A erroneously state that mice received “Buccal IM+EP” instead of “Buccal injection+EP.” The corrected figures appear here.

**FIG 4:**
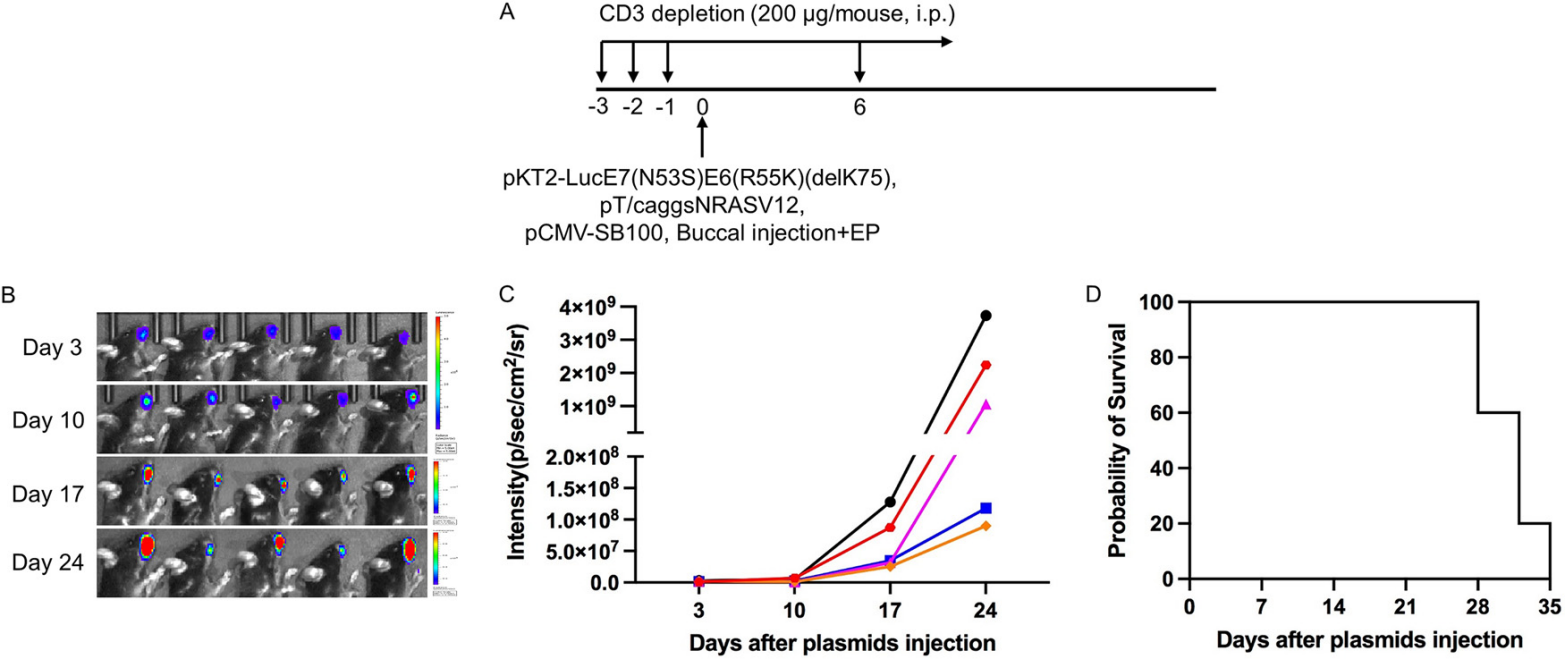
Generation of spontaneous HPV16 E6E7-expressing oral sarcoma model in HLA-A2 (AAD) transgenic mice.

**FIG 6:**
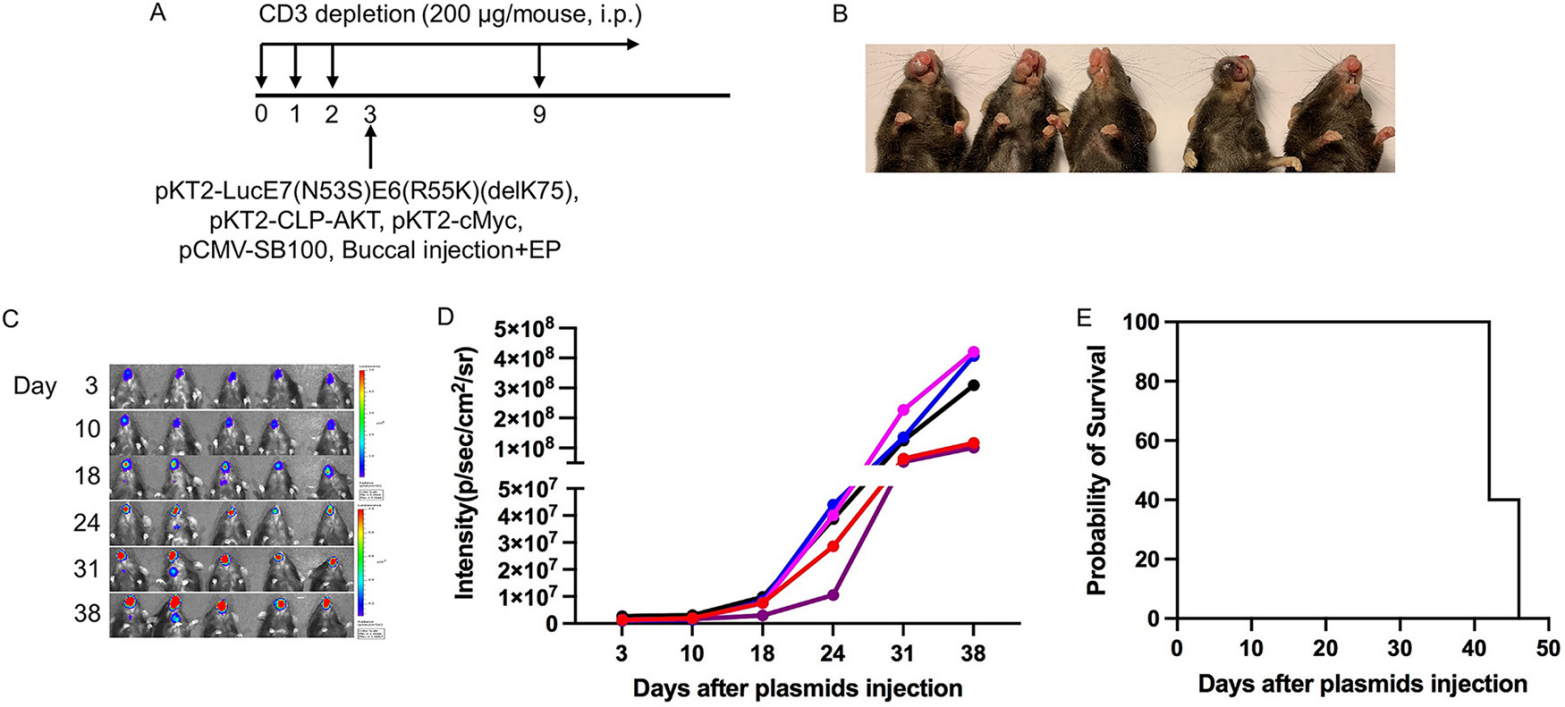
Generation of spontaneous HPV16 E6E7-expressing oral/pharyngeal carcinoma model in HLA-A2 (AAD) transgenic mice.

